# Enhanced resistive switching characteristics in Pt/BaTiO_3_/ITO structures through insertion of HfO_2_:Al_2_O_3_ (HAO) dielectric thin layer

**DOI:** 10.1038/srep46350

**Published:** 2017-04-11

**Authors:** J. P. B. Silva, F. L. Faita, K. Kamakshi, K. C. Sekhar, J. Agostinho Moreira, A. Almeida, M. Pereira, A. A. Pasa, M. J. M. Gomes

**Affiliations:** 1 Centre of Physics, University of Minho, Campus de Gualtar, 4710-057 Braga, Portugal; 2 IFIMUP and IN-Institute of Nanoscience and Nanotechnology, Departamento de Física e Astronomia, Faculdade de Ciências da Universidade do Porto, Rua do Campo Alegre 687, 4169-007 Porto, Portugal; 3 Departamento de Física, Universidade Federal de Santa Catarina, Campus Trindade, 88040-900 Florianópolis, SC, Brazil; 4 Department of Physics, Madanapalle Institute of Technology & Science, Madanapalle, 517325, Andhra Pradesh, India; 5 Department of Physics, Central University of Tamil Nadu, Thiruvarur, 610 101, India

## Abstract

An enhanced resistive switching (RS) effect is observed in Pt/BaTiO_3_(BTO)/ITO ferroelectric structures when a thin HfO_2_:Al_2_O_3_ (HAO) dielectric layer is inserted between Pt and BTO. The P-E hysteresis loops reveal the ferroelectric nature of both Pt/BTO/ITO and Pt/HAO/BTO/ITO structures. The relation between the RS and the polarization reversal is investigated at various temperatures in the Pt/HAO/BTO/ITO structure. It is found that the polarization reversal induces a barrier variation in the Pt/HAO/BTO interface and causes enhanced RS, which is suppressed at Curie temperature (*T*_*c*_ = 140 °C). Furthermore, the Pt/HAO/BTO/ITO structures show promising endurance characteristics, with a RS ratio >10^3^ after 10^9^ switching cycles, that make them potential candidates for resistive switching memory devices. By combining ferroelectric and dielectric layers this work provides an efficient way for developing highly efficient ferroelectric-based RS memory devices.

Resistive random access memories (RRAMs) have drawn particular attention for the next generation of non-volatile memories due to their unique advantages such as excellent scalability, fast switching, high integration density and good compatibility with the current complementary metal oxide semiconductor (CMOS) technology^1^,^2^. The intrinsic physical phenomenon behind RRAMs is resistive switching (RS), which means that the device can be freely programmed into a high resistance state (HRS) or a low resistance state (LRS) under external electric field.

A wide variety of materials such as perovskite-type oxides, ferroelectric oxides and binary transition metal oxides (TMOs) have been extensively investigated^1^. However, RS effect in most of these materials is based on a certain type of defect mediated phenomenon^2^. But the lack in a precise control of the switching behavior based on charged defect migration hinders them for the practical applications. On the other hand, RRAMs based on ferroelectric thin films sandwiched between two asymmetric electrodes have been attracting considerable attention recently. For these structures, low and high resistance states can be tuned via intrinsic switching of ferroelectric polarization without invoking the charged defect migration like in pure semiconductors and dielectrics^3^,^4^.

It is well known that the Schottky like barrier generates an asymmetric potential distribution in ferroelectric layer and causes the ferroelectric RS effect^5^,^6^. Recent theoretical studies show that the insertion of a very thin dielectric layer at one of the interfaces can significantly enhances the RS effect^7^. The metal-dielectric-ferroelectric-semiconductor structures will allow artificially the tailoring of the asymmetric potential distribution at metal-ferroelectric interface, through the insertion of an ultrathin dielectric layer at this interface^8^. In this paper, we made an attempt to study the RS effect in this kind of configuration.

In this context, barium titanate (BaTiO_3_-BTO) is chosen as the ferroelectric material due to its eco-friendly nature, excellent spontaneous polarization, as large as 26 μC/cm^2^, moderate coercive field (≈1 kV/cm) and ferroelectric transition temperature T_c_ well above room temperature (T_c_~120 °C)^9^,^10^,^11^. From all high-k dielectrics, hafnium oxide (HfO_2_) has been the predominant choice for the gate dielectric in metal-oxide-semiconductor (CMOS) devices, dynamic random access memory (DRAM) capacitors, and blocking insulator in Si-oxide-nitride-oxide-silicon (SONOS)-type flash memory cells^12^,^13^. Although HfO_2_ is well recognized as a potential candidate for RRAM applications, the resistive switching effect is mostly based on charge defect migration^14^. Furthermore, it has been shown that hafnium aluminates (HfO_2_:Al_2_O_3_-HAO) exhibit improved properties such as high thermal stability, large band gap and high barrier for oxygen diffusion^12^,^15^,^16^. Yet, there are only few reports on the RS characteristics of dielectric-ferroelectric structures, and the polarization coupling at Pt/HAO/BTO heterojunction is not reported. Further, the polarization fatigue effect on RS characteristics in these structures is not established yet. Taking into account that Joule heating can result in unrecoverable behavior of the devices^17^, the study of the Pt/HAO/BTO heterojunction is of particular interest due to the aforementioned characteristics of HAO.

In this work, a detailed structural analysis, by grazing incidence x-ray diffraction (GIXRD) and transmission electron microscopy (TEM), and its impact on the ferroelectric and electrical properties of Pt/BTO/ITO and Pt/HAO/BTO/ITO structures is presented and discussed, in order to unravel the effect of including a HAO thin layer on resistive switching. In order to assess the performance of Pt/HAO/BTO/ITO structures for non-volatile memory applications, fatigue studies are also carried out.

## Results and Discussion

**Figure 1:**
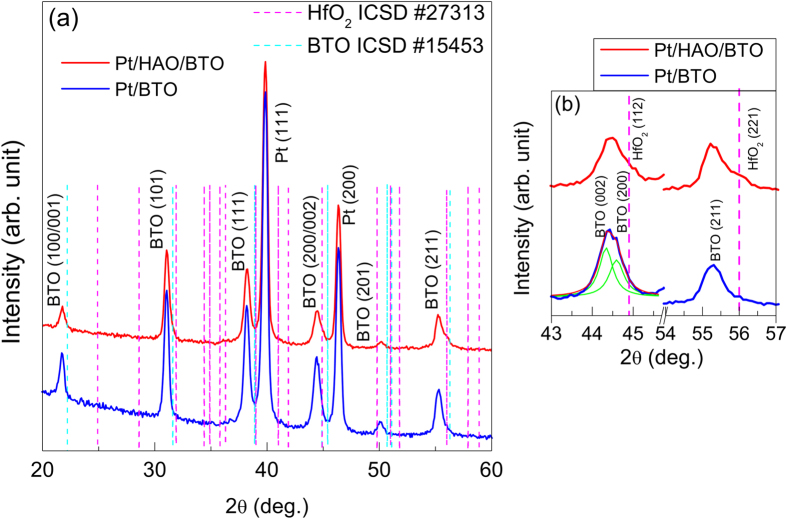
(**a**) GIXRD patterns of the Pt/BTO and Pt/HAO/BTO structures; (**b**) extended GIXRD scans.

The GIXRD patterns of Pt/BTO and Pt/HAO/BTO samples are illustrated in Fig. 1(a). The X-ray diffraction (XRD) patterns using Bragg-Brentano geometry (θ–2θ) for both samples are given in Supplementary Fig. S1. These results are similar to those obtained from the GIXRD.

Both GIXRD patterns exhibit the characteristic Bragg peaks of the tetragonal perovskite phase of BTO, without any secondary phase^9^. These reflections were assigned to the crystallographic planes according to ICSD card #15453, which are marked with blue lines. The presence of the monoclinic phase of HfO_2_ was confirmed in the Pt/HAO/BTO structure by the presence of weak diffraction peaks at 2θ ≈ 45.0° and 56.0° corresponding to the HfO_2_ (*112*) and HfO_2_ (*221*) reflections^18^. These reflections were assigned to the crystallographic planes according to ICSD card #27313, which are marked with pink lines in Fig 1(a) and (b). Usually, the crystallization of Al_2_O_3_ and HfO_2_ appears above 1000 °C and below 500 °C, respectively^19^. The absence of Al_2_O_3_ peaks in XRD could be due to its amorphous nature. The presence of clear splitting of the BTO (*200*) peak around 2θ ≈ 44.5° in Pt/BTO confirms the tetragonal symmetry of the BTO layer at room temperature^20^. The extended scans of GIXRD around 2θ ≈ 44.5° are shown in Fig. 1(b). The splitting of the BTO (*200*) peak is not visible in Pt/HAO/BTO structures, as it is superposed, by a weak shoulder peak around 2θ ≈ 45.0°, assigned to the presence of the HfO_2_ (*112*) reflection.

In addition, compared to the position of the Bragg peaks of the ICSD card, the reflections of BTO in both structures are shifted to lower 2θ values, which could be due to the presence of tensile stress, appearing during the cooling after the annealing treatment, due to the thermal expansion coefficient difference between film and substrate^21^,^22^. The thermal stress and strain are given by^23^:


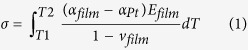



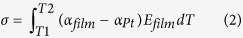


where α_film_ and α_Pt_ are the thermal expansion coefficients of the BTO film and Pt layer, respectively, and E_film_ and υ_film_ are the elastic modulus and Poisson’s ratio, respectively. The thermal expansion coefficient for Pt is 8.9 × 10^−6^ K^−1^ and 10.5 × 10^−6^ K^−1^, at room temperature and at the annealing temperature (650 °C)^24^, while, for BaTiO_3_, these coefficients are equal to 6.0 × 10^−6^ °C^−1^ and 14.2 × 10^−6^ °C^−1^, respectively^25^,^26^. By considering the elastic modulus and Poisson’s ratio equal to 128 GPa^27^ and 0.35^28^, the thermal stress and strain were calculated to be 0.67 GPa and 0.3%, respectively.

It is known that the lattice parameters of BTO are strongly influenced by the existence of stress. Therefore, the lattice parameters “*a*” and “*c*” are calculated (using the XRD pattern information – see Supplementary Fig. S1) by the least square method using the positions of major peaks, such as (*100*)/(*001*), (*101*), (*111*), (*200/002*), (*201*) and (*211*), and the results are shown in Table 1, along with the tetragonality value (c/a ratio). It is observed that the tetragonality value (≈1.003) in both structures is lower than the value for the powdered polycrystalline BTO (≈1.011)^29^. Moreover, we should stress out that misfit strain cannot explain the tensile stress, since the a-parameter for Pt (*a*_*Pt*_ = 3.908 Å) is lower than for BTO film. For a qualitative characterization of the films orientation, the following expression was used^30^:


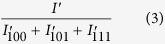


where I′ is equal to I/I*. Here, I and I* are the intensities of a particular reflection in the BTO film and powdered polycrystalline (ICSD card #15453), respectively. From the calculated intensity ratio for both structures, shown in Table 1, one observes that the BTO layers have the (*111*) preferred orientation. In fact, the (*101*) peak is much more intense than (*111*) in bulk BTO. The I_(*101*)_/I_(111)_ ratio calculated using the data from ICSD card #15453 is equal to 3.1, while in the BTO layers it is equal to 1.1. The small lattice mismatch between BTO and Pt (*111*) helps BTO to grow in the same orientation as that of Pt^31^.

**Figure 2:**
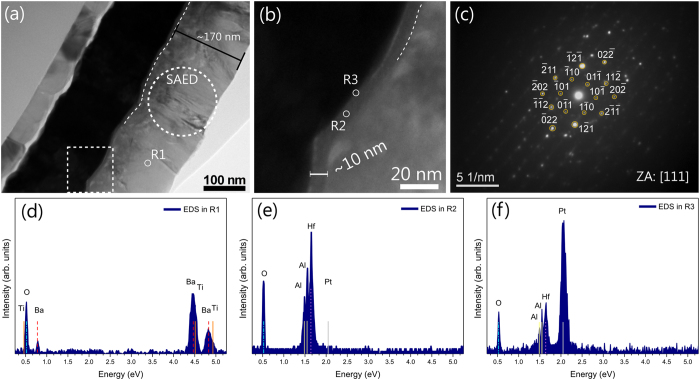
(**a**) Cross-section TEM image of the SiO_2_/TiO_2_/Pt/HAO/BTO structures; (**b**) Zoom of the Pt/HAO/BTO interfaces; (**c**) SAED pattern obtained from circular region; (**d**)**-**(**f**) EDS on the different parts of the structure marked as R1, R2 and R3 in Figs (**a**,**b**).

The Pt/HAO/BTO structure was also investigated by transmission electron microscopy (TEM). Figure 2(a) shows an overview cross-section image of the Pt/HAO/BTO structure. The thicknesses of the HAO and BTO layers are found to be around 10 nm and 170 nm, respectively. It is also observed that the BTO layer shows a dense columnar structure. Figure 2(b) shows a zoom of the structures with HAO layer in highlight between the bottom and top interfaces. The Pt/HAO and HAO/BTO interfaces are slightly wavy and the thickness of the HAO layer is not uniform. The dashed line highlights a region with a reduced thickness in HAO layer of 7 nm. However, the overall structural integrity of the HAO layer is intact and there is no sign of Pt diffusion. The BTO layer shows a characteristic contrast of dense columnar structure, suggesting a typical polycrystalline growth with a considerable degree in preferential orientation. To clarify this, selected area electron diffraction (SAED) was performed in the BTO layer, and the indexed pattern is shown in Fig. 2(c). The indexation was performed using the Jems^®^ software, with ICSD card #15453 as theoretical crystalline information (a = 3.9945 Å and c = 4.0335 Å). The cell parameters obtained from GIXRD (a = 4.067 Å and c = 4.077 Å) and orientation [111] as the zone axis (ZA) were applied in the indexation to obtain a better fit. All recorded SAED’s patterns showed domains with a polycrystalline diffraction and predominant crystalline orientation. These results corroborate the polycrystalline character and the considerable degree of crystalline orientation, already evidenced by the XRD data.

The EDS (using a 3 nm spot size electron beam) was performed on the different parts of the structure (marked as R1 in Fig. 2(a) and R2 and R3 in Fig. 2(b)) to understand the chemical composition of HfO_2_:Al_2_O_3_ and to explore the possibility of diffusion and/or the composition variation with the depth of the film, and is shown in Fig. 2(d–f). No diffusion could be detected in the resolution limit of the equipment and the EDS spectra exhibited the characteristic peaks of elements Hf and Al, confirming the presence of these elements in the HAO layer. Moreover, the Ba/Ti ratio in the BTO layer was estimated from EDS and is equal to 1.02, which is close to the expected ratio in BTO.

**Figure 3:**
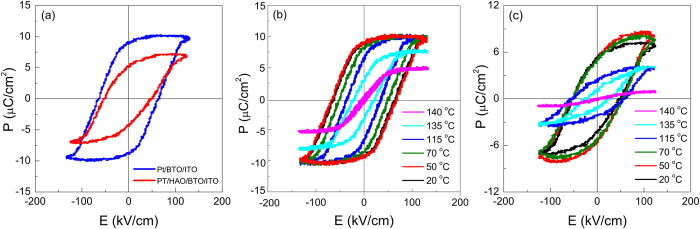
(**a**) *P-E* hysteresis loops of Pt/BTO/ITO and Pt/HAO/BTO/ITO structures at room temperature; (**b**) and (**c**) *P-E* temperature dependence for the Pt/BTO/ITO and Pt/HAO/BTO/ITO structures, respectively.

Figure 3(a) shows the P-E hysteresis loops of Pt/BTO/ITO and Pt/HAO/BTO/ITO structures at room temperature. The values of positive and negative remnant polarization (±*P*_*r*_), spontaneous polarization (±*P*_*s*_), and coercive field (±*E*_*c*_) observed in both structures are presented in Table 2. The Pt/BTO/ITO structure exhibits rather symmetric hysteresis loops with an average *P*_*r*_ of 8.9 μC/cm^2^, *P*_*s*_ of 9.7 μC/cm^2^ and *E*_*c*_ of 64.2 kV/cm. The obtained *P*_*r*_ value is higher than the values found in literature for polycrystalline BTO thin films grown by pulsed laser deposition (PLD) method^20^, which can be explained by the highly (*111*)-oriented texture of BTO films. This is in good agreement with literature^31^,^32^. Usually, the epitaxial films exhibit higher polarization than polycrystalline films since there are no grain boundaries to obstruct the domain orientations^33^. The Pt/HAO/BTO/ITO structures display a hysteresis loop with an average *P*_*r*_ of 4.9 μC/cm^2^, *P*_*s*_ of 6.9 μC/cm^2^ and *E*_*c*_ of 52 kV/cm.

It should be noted that the P-E loop in case of Pt/HAO/BTO/ITO structures is slightly asymmetric. This asymmetry is due to an internal build-in electric field, with value ≈3 kV/cm, created by the charge accumulation at the dielectric/ferroelectric interface^34^. In addition, the decrease of *P*_*r*_, *P*_*s*_ and *E*_*c*_ in Pt/HAO/BTO/ITO structure is consistent with the predictions from the theoretical model presented by A. K. Tagantsev *et al*. for “ferroelectric film + thin dielectric layer”, which predicts that the three parameters are decreasing functions of d/L, where d and L are the thicknesses of the passive dielectric layer and ferroelectric film, respectively^35^. Figure 3(b) and (c) depict the P-E loops of Pt/BTO/ITO and Pt/HAO/BTO/ITO structures at different fixed temperatures, in the temperature range 20 °C–140 °C. The hysteresis loops become slimmer with the increasing temperature and vanish when the temperature reaches 140 °C. This confirms the ferroelectric to paraelectric phase transition, whose temperature is larger than the Curie temperature (*T*_c_ = 120 °C) reported for BTO ceramics^9^. The increase of *T*_c_ is due to the presence of tensile stress^36^, owing to the thermal expansion coefficient difference between film and substrate, as it is evidenced by XRD analysis. The moderate increase in transition temperatures is consistent with the Landau-Ginsburg-Devonshire theory for polydomain thin films with residual misfit strain less than 0.2%^37^. In our case, the increase of *T*_*c*_ is predominantly due to the thermal tensile strain, which was calculated from the XRD analysis and is equal to 0.3%.

**Figure 4:**
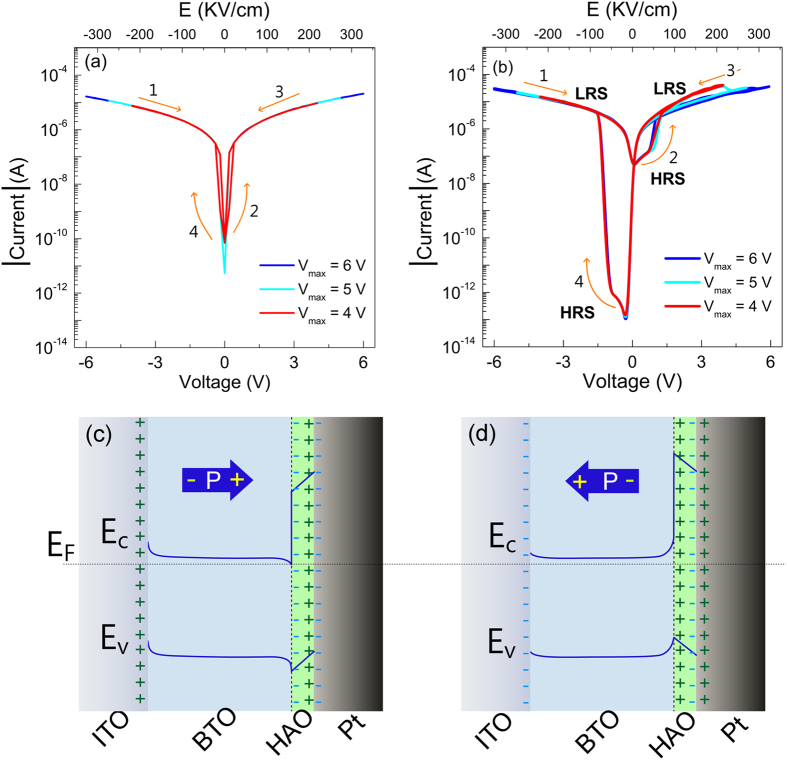
I-V characteristics of (**a**) Pt/BTO/ITO and (**b**) Pt/HAO/BTO/ITO structures. Schematic charge distribution and energy-band diagrams for Pt/HAO/BTO/ITO as (**c**) a “reverse diode”, and (**d**) as a “forward diode”.

**Figure 5:**
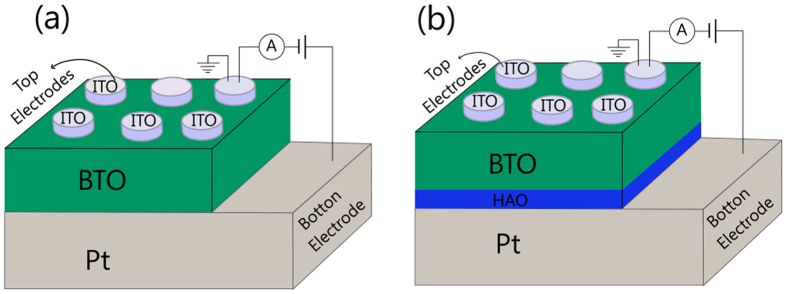
Schematic diagrams of the experimental test circuit for the (**a**) Pt/BTO/ITO and (**b**) Pt/HAO/BTO/ITO structures.

Figure 4(a) and (b) depict the room temperature I-V characteristics of Pt/BTO/ITO structures with and without HAO thin dielectric layer, wherein I stands for the modulus of the current. In fact, for the negative dc voltage, the actual current has the opposite signal. Since BTO films are assumed to be n-type semiconductors, the measured current I results from the naturally produced oxygen vacancies, which act as electron donors^17^,^38^. The dc voltage V was applied on the bottom Pt electrode with the top ITO electrode as the ground, as it is schematically shown in Fig. 5. The dc voltage was first swept from −V_max_ to +V_max_, and then in the reverse direction.

During the measurements, different V_max_ values were applied in order to investigate the dependence of the leakage current on the amplitude of V_max_. The work functions of polycrystalline Pt and ITO are 5.6 eV and 4.5 eV, respectively^30^,^39^. This leads to an electric potential asymmetry, with a rectification ratio of 1.3, in the I-V curves of the Pt/BTO/ITO structures, as one can see in Fig. 4(a). Although the growth conditions and electrode configurations were similar, significant differences can be observed in I-V curves when a thin layer of HAO is inserted between Pt/BTO. In spite of the similar magnitude of current at ±V_max_ in both samples, the hysteresis effect is much more pronounced in Pt/HAO/BTO/ITO structures. Usually, the insertion of HAO may lower the current. However, the possible explanation for the similar magnitude of current at V_max_ in both samples is the existence of preferential electron tunneling at hot spot sites^40^. These hot spots can correspond to points of reduced thickness in an overall continuous barrier. As it was mentioned before, the thickness of the HAO layer is not uniform, so, this may give rise to hot spot sites. As illustrated in Fig. 4(b), all the I-V curves with different V_max_ show the RS effect. The I-V characteristics with V_max_ = 4 V are also shown in Supplementary Fig. S2. They exhibit the bipolar resistive switching without any electroforming process. This is an advantage with ferroelectric resistive switching memories compared to the one based on oxides materials where the electroforming is a necessary step. The device displays the two different states in the voltage region of 0 to ±1. The resistance of the two states, such as low resistance state (LRS) and high resistance state (HRS), was read-out at −0.4 V and the RS ratio [R_HRS_/R_LRS_] was found to be ≈5 × 10^6^, which is at least two orders of magnitude higher than the ones reported in literature for other ferroelectric-dielectric structures, with different dielectric layers, such as HfO_2_ and LaFeO_3_^8^,^41^,^42^. Furthermore, the RS behavior in the negative voltage region is more expressive than in the positive voltage region, due to the asymmetric interface^12^,^43^. It is further observed that the switching from HRS to LRS occurred at threshold voltage of ±1 V, as expected in ferroelectric Schottky diodes^44^. The switching field (E_s_) = 56 ± 6 kV/cm, corresponding to 1 V, is consistent with the coercive voltages for polarization switching (E_c_) = 52 ± 1 kV/cm. We can switch our devices from one state to the other as follows: if we apply the −0.4 V at a certain instant of time and found that the device is in HRS mode, then the device can be set into LRS mode by applying a voltage less than −1 V (as we observed in Fig. 4(b)). If the device is in LRS mode at time of test, we can set the device in HRS mode, at −0.4 V, by applying a voltage greater than +1 V^3^.

Usually, the RS effect in ferroelectric materials is attributed to the ferroelectric polarization modulation of Schottky barrier^5^. As compared to Pt metal electrodes, the screening effect of ITO on the bound charges is less efficient due to its lower carrier concentration. This leads to a minor potential distribution at BTO/ITO interface and causes a small RS effect in Pt/BTO/ITO structures, as shown in Fig. 4(a). However, RS effect is significantly enhanced, as shown in Fig. 4(b), when a thin layer of HAO is inserted between Pt and BTO, and this can be understood based on the experimental data, the schematic charge distribution and energy-band alignment of constituent materials. In the Pt/HAO/BTO/ITO structures, HAO layer separates the screening charges in the electrode from the polarization charge in BTO layer and this leads to a finite potential distribution in the HAO dielectric layer^8^,^45^.

Before starting to discuss the mechanisms driving the I-V behavior of Pt/HAO/BTO/ITO structures, it is worth looking into detail to the role of the charge configurations of Fig. 4(c) and (d). As it will be seen below, these configurations have the characteristics of a diode, and they will be called from now on the “reverse diode” and “forward diode”, wherein reverse and forward stand for polarization oriented to the Pt electrode (downward direction) and vice-versa (upward direction), respectively. Coming now to the analysis of Fig. 4(b), it is observed that under a negative dc voltage, the ferroelectric polarization in BTO points towards the Pt electrode (Fig. 4(c)), and does not change its direction until a positive dc voltage higher than the coercive field is applied. The positive polarization charges induce negative charges at the HAO layer close to BTO, and thus, induced positive charges emerge in the HAO layer at HAO/Pt interface, which attracts electrons towards the interface, as shown in Fig. 4(c). Whereas for the negative range of the dc voltage, the “reverse diode” acts like a conducting one (LRS state), for the positive range below to the coercive field it plays the role of a non-conducting diode (HRS state). For positive dc voltages above the coercive field both the polarization and the charge distribution of the HAO layer reverse their direction and consequently the conducting “forward diode” (Fig. 4(d)) stabilizes the LRS state. The behavior for a reversed sweep of the dc voltage can be understood in a similar way, by just swapping “reverse diode” for “forward diode”. Therefore, the RS effect in Pt/HAO/BTO/ITO structures can be well explained based on the modulation of the potential distribution at the HAO/BTO interface via ferroelectric polarization reversal^8^,^17^,^45^.

**Figure 6:**
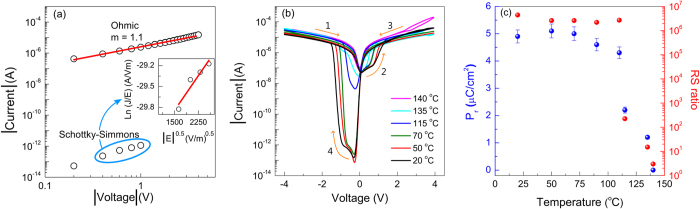
(**a**) Linear fitting to the LRS state for Pt/HAO/BTO/ITO structures on a logarithmic scale for the negative bias region. The inset shows the Schottky-Simmons plot of ln(J/E) versus E^0.5^ to the HRS state, in the negative voltage region −0.4 to −1.0 V; (**b**) I-V temperature dependence for the Pt/HAO/BTO/ITO structures and (**c**) The average remnant polarization (*P*_*r*_) and RS ratio as a function of the temperature for the Pt/HAO/BTO/ITO structures.

In order to understand the conduction mechanism in the Pt/HAO/BTO/ITO structures, several leakage models, such as the space-charge-limited current (SCLC), Poole-Frenkel (PF), Schottky emission (SE), the hopping conduction, or the Schottky–Simmons were considered to interpret the RS behavior. In the present devices, the fitting with PF and SE models to HRS data gave non-physical values of refractive index (*n*) of 1.42 and 0.97, respectively. However, the experimental data can be well scaled with ln(*J/E) vs. E*^0.5^ which can be associated with the Schottky–Simmons model. According to this model, the current density is as follows^30^:


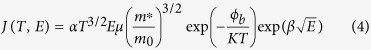


which takes into account both the carriers density at the potential barrier maximum near the interface and their mobility which is a bulk property. As can be seen in the inset of the Fig. 6(a), the data handling in the case of Schottky–Simmons gives the appropriate values of n = 2.23, which is in a good agreement with the reported values for BTO materials^46^. On the other hand, the LRS state follows simply the Ohmic behavior as shown in Fig. 6(a).

The quality of the fittings of LRS by Ohm law (R^2^ = 0.99) and HRS by Schottky-Simmons emission (R^2^ = 0.92) is a clear signature of an interface based mechanism^47^. This suggests that the RS is controlled by the direction of the polarization. However, it is also necessary to check if the observed RS has (dominant) contributions from any filamentary processes.

To distinguish between the RS controlled by the direction of the polarization and a filamentary process, we have performed the I-V curve for the Pt/HAO/ITO structure which is shown in Supplementary Fig. S3. No RS effect is observed in these structures. In fact, we need an electroforming process to initiate the RS effect in HAO based memories. Electroforming process induces the high density of defects and causes the RS effect due the formation of filaments via defects. According to literature, the switching field in HfO_2_-based structures is around 1000 kV/cm^12^,^48^,^49^, which is at least 3 times higher than the field applied in our structures. Therefore, the applied field is not enough to initiate any unintentional electroforming process in HAO. Besides that, we have grown defect-free HAO thin films as described in ref. 15. Therefore, the enhancement in RS through the insertion of HAO can be attributed to finite potential distribution in the HAO dielectric layer. To further confirm it we have studied the temperature dependence of the I-V characteristics for Pt/HAO/BTO/ITO structures. Figure 6(b) shows I-V curves of the Pt/HAO/BTO/ITO structures measured at different temperatures in the range of 20 °C to 140 °C. It is seen that the RS ratio and RS window change significantly with the temperature. Figure 6(c) shows a quantitative analysis of the average *P*_*r*_ magnitude and RS ratio as a function of temperature. The average *P*_*r*_ and RS ratio remains almost constant with increasing temperature up to 110 °C and falls down with further increase in temperature. The disappearance of the RS effect and the closure of the P-E loop at temperature above 140 °C are due to the ferroelectric-paraelectric phase transition. Therefore, the simultaneous disappearance of the RS effect and *P*_*r*_ and the similitude of coercive field (E_c_) = 52 ± 1 kV/cm and switching field (E_s_) = 56 ± 6 kV/cm confirm the strong coupling between the resistive switching and the polarization switching^50^. Usually, the leakage currents in BTO films are attributed to the inevitable oxygen vacancies and, thus, BTO is assumed as n-type semiconductor. However, oxygen vacancies do not play an essential role in disclosing the RS effect, as it does not occur in Pt/BTO/ITO. Contrarily, when HAO is inserted between Pt and BTO, an enhanced RS effect is observed, which can be attributed to the emergence, at the BTO-HAO interface, of a charge coupling mechanism mediated by a high energy barrier. The oxygen vacancies in BTO layers can move along the polarization direction and participate in barrier modulation, but its contribution to RS effect seems to be nominal, as the RS effect disappears when the device is set in paraelectric state.

The built-in potential change by the ferroelectric polarization can be calculated from Δϕ_bi_^P^ = ϕ_bi_^|^ − ϕ_bi_ = ±Pδ/ε_0_ε_s_, where ϕ_bi_ is the built-in potential without contribution from polarization, ϕ_bi_^|^ is the built-in potential with contribution from polarization, P is the ferroelectric polarization, ε_0_ is the free space permittivity, ε_s_ is the static dielectric permittivity, and δ is the thickness of a interface layer between the surface polarization charge and the HAO layer^17^. The value of δ is of the order of a unit cell (1 nm)^17^. To estimate the variation in the built-in potential for the HAO/BTO interface, the polarization values obtained from P-E loops at different temperatures and the measured frequency (1 kHz) electric permittivity (no dielectric dispersion was observed at lower frequencies) were used. The variation in Δϕ_bi_^P^ was estimated to be ±0.2, ±0.1 and ±0.0 V for the different temperatures of 20, 135, and 140 °C, respectively. Therefore, one can understand that large polarization induces a larger built-in potential change and consequently a higher RS ratio.

**Figure 7:**
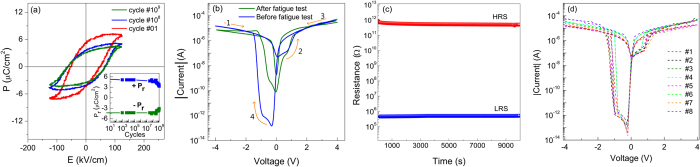
(**a**) *P-E* hysteresis loops of Pt/HAO/BTO/ITO structures at different cycles. The inset shows the plot of +*P*_*r*_ and −*P*_*r*_ versus number of switching cycles; (**b**) I-V characteristics of Pt/HAO/BTO/ITO structures before and after the fatigue test; (**c**) Retention characteristics of the Pt/HAO/BTO/ITO structures and (**d**) I-V curves collected from randomly chosen devices.

To evaluate the possible application in memory devices, the endurance and retention characteristics of the Pt/HAO/BTO/ITO structures were investigated. Figure 7(a) depicts the P-E loops after several polarization cycles and the inset shows the positive and negative remnant polarization versus reversal cycles.

It is noticeable that the *P*_*r*_ value was decreased by 33% after performing 10^9^ reversal cycles. Similar decrease of *P*_*r*_ was observed in Pt/BTO/ITO structure (not shown here) and therefore, the tensile stress, as suggested by the XRD analysis, might be responsible for the observed decline. Moreover, similar decrease in the *P*_*r*_ value was observed in PZT thin films under a tensile stress of 0.2%^51^. The I-V characteristics of the Pt/HAO/BTO/ITO structures after the fatigue test (10^9^ reversal cycles) were also investigated. Figure 7(b) shows the I-V curves before and after fatigue tests and it is clearly observed that even after 10^9^ switching cycles the structures exhibit RS characteristics, with a RS ratio >10^3^. The decrease in the RS ratio is caused by the decline in *P*_*r*_, which leads to a weaker modulation of the potential distribution at the HAO/BTO interface. However, the RS ratio after 10^9^ switching cycles is still even higher than the values found in the literature, before the fatigue test, for structures such as LaFeO_3_/Bi_1−δ_FeO_3_ sandwiched between SrRuO_3_ electrodes (RS ratio ≈10^2^) and Au/BiFeO_3_/SrRuO_3_ (RS ratio ≈10^2^)^8^,^45^,^52^.

For the retention tests, a reading voltage of −0.6 V was applied and the device was found to be in LRS state. Then, −0.6 V was periodically applied with the interval of 1 s, and its variation with the time is shown in Fig. 7(c). The device was set into HRS mode by applying sweeping voltage to +4.0 V and the HRS retention test was performed in the same way, and the result is also shown in Fig. 7(c). Both LRS and HRS states are stable in time. Several typical I-V curves collected from randomly chosen devices are given in Fig. 7(d). I-V curves revealed similar RS characteristics. This evidences that the structures display a reliable bipolar resistive switching behavior and thus, the device exhibits promising non-volatile characteristics.

## Conclusions

In this work, it was demonstrated that the insertion of a Al_2_O_3_-HfO_2_ thin dielectric layer at the Pt/BTO interface enhances resistive switching in Pt/BTO/ITO structures. The coupling between the resistive switching and the ferroelectric polarization reversal in the Pt/HAO/BTO/ITO structures was investigated at different temperatures. The analysis of the temperature dependencies of the P_r_ and the RS ratio allows concluding that the ferroelectric polarization reversal induces the RS effect in the structures. Therefore, the RS effect with a ratio >10^6^ was attributed to the modulation of the potential barrier at HAO/BTO interface via ferroelectric polarization reversal. The endurance tests revealed that the Pt/HAO/BTO/ITO structures display a RS ratio >10^3^ after 10^9^ switching cycles, which make them attractive for memory devices. So, the present work provides a guide for designing the next generation of non-volatile memory devices with low-power and high RS ratio by using ferroelectric-based structures.

## Methods

HfO_2_:Al_2_O_3_ and BaTiO_3_ thin films were grown using a commercially available HfO_2_:Al_2_O_3_ = 1:1 (GoodFellow, 99.9%) and BaTiO_3_ (Neyco, 99.9%) targets by ion beam sputter deposition (IBSD) technique with a multi-target carousel system that allowed depositing both layers without breaking the vacuum. The chamber was evacuated down to a low pressure of 1 × 10^−6^ mbar prior to the deposition. During the deposition, the substrate was kept at a temperature of 330 °C and at a distance of 87.3 mm from the target. The gas pressure inside the chamber was kept constant at 3.4 × 10^−4^ mbar. A gas flow of 6.0 ml/min of Ar + 2.0 ml/min of O_2_ was introduced into the ion beam gun and the atoms were ionized in the ion source with an rf-power of 120 W. The ions beam was further accelerated to 900 V the ion beam current was found to be 31 mA. Two types of samples, namely BTO and HAO/BTO, were grown by depositing a 170 nm BTO (BTO single layer), and a 10 nm thick HfO_2_:Al_2_O_3_ and 170 nm BTO layers (HAO/BTO bilayer structure), on top of Si/SiO_2_/TiO_2_/Pt substrates. Subsequently, an annealing was performed in vacuum (≈2.0 × 10^−5^ mbar) at 650 °C for 30 min in order to improve the crystallinity of the structures. Moreover, a low O_2_/Ar ratio was used in the deposition to obtain *n*-type BTO films^38^,^53^.

The structures were structurally characterized by grazing incidence x-ray diffraction (GIXRD) with a PanAnalytical Xpert PRO multi-purpose diffractometer using Cu-Kα radiation (λ = 1.54056 Å) and ω = 1.5°. Cross-sectional microstructure of the HAO/BTO structures was studied by transmission electron microscopy (TEM) using a Jeol JEM-2100 microscope operated at 200 kV. For TEM investigations the samples were prepared by conventional cross-section method. The images and SAED were collected keeping the sample oriented on the [110]Si as zone axis and the analyzes were performed with DigitalMicrograph-Gatan^®^ and Jems^®^ softwares, respectively. Energy dispersive spectroscopy (EDS) was performed using a Thermo Scientific system attached to the TEM for evaluating the atomic composition of elements present in the layers. Furthermore, TEM measurements were done in a virgin sample before any electric stress.

For the electrical characterization top indium tin oxide (ITO) electrodes, with a diameter of 1 mm, were deposited by IBSD on the upper surface of BTO films using a commercially available 90 wt.% In_2_O_3_ −10 wt.% SnO_2_ target (Kurt J. Lesker, 99.99%). During the deposition, the substrate was kept at a temperature of 100 °C and the gas pressure inside the chamber was kept constant at 3.4 × 10^−4^ mbar. A gas flow of 7.7 ml/min of Ar + 0.3 ml/min of O_2_ was introduced into the ion beam gun and the atoms were ionized in the ion source with an rf-power of 50 W. The ferroelectric hysteresis loops (P–E) of Pt/BTO/ITO and Pt/HAO/BTO/ITO structures were measured with a modified Sawyer-Tower circuit using a sinusoidal signal at a frequency of 1 kHz. Current-voltage (I-V) characteristics were measured using a Keithley 617 programmable electrometer. In all the electrical measurements, the temperature was measured with an accuracy of 0.1 °C.

## Additional Information

**How to cite this article**: Silva, J. P. B. *et al*. Enhanced resistive switching characteristics in Pt/BaTiO_3_/ITO structures through insertion of HfO_2_:Al_2_O_3_ (HAO) dielectric thin layer. *Sci. Rep.* **7**, 46350; doi: 10.1038/srep46350 (2017).

**Publisher's note:** Springer Nature remains neutral with regard to jurisdictional claims in published maps and institutional affiliations.

## Supplementary Material

Supplementary Information

## Figures and Tables

**Table 1:**
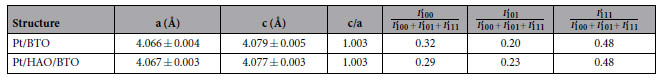
Lattice parameters, tetragonality (c/a ratio) and relative intensity ratio of peaks in BTO layers in different structures.

**Table 2:** Positive and negative remnant and spontaneous polarization (+*P*_*r*_, −*P*_*r*_, +*P*_*s*,_ and −*P*_*s*_) and coercive field (+*E*_*c*_ and −*E*_*c*_) values.

Structure	+P_r_ (μC/cm^2^)	−P_r_ (μC/cm^2^)	+P_s_ (μC/cm^2^)	−P_s_ (μC/cm^2^)	+E_c_ (kV/cm)	−E_c_ (kV/cm)
Pt/BTO/ITO	9.0 ± 0.1	−8.8 ± 0.1	10.0 ± 0.1	−9.4 ± 0.1	65 ± 1	−64 ± 1
Pt/HAO/BTO/ITO	5.4 ± 0.1	−4.3 ± 0.1	6.8 ± 0.1	−6.9 ± 0.1	50 ± 1	−54 ± 1
