# Supplementary material for: Enhanced resistive switching characteristics in Pt/BaTiO3/ITO structures through insertion of HfO2:Al2O3 (HAO) dielectric thin layer
Source: Sci Rep. 2017 Apr 11;7:46350. doi: 10.1038/srep46350 (PMC5387719; doi:10.1038/srep46350)
Supplement: Supplementary Information [file srep46350-s1.pdf]

Supplementary Information

Enhanced resistive switching characteristics in Pt/$BaTiO_3$/ITO structures through insertion of HfO_2:Al_2O_3 (HAO) dielectric thin layer

J. P. B. Silva[1,2,*], F. L. Faita[1,3], K. Kamakshi[1,2,4], K. C. Sekhar[1,5], J. Agostinho Moreira[2], A. Almeida[2], M. Pereira[1], A. A. Pasa[3],and M. J. M. Gomes[1]

[1]Centre of Physics, University of Minho, Campus de Gualtar, 4710-057 Braga, Portugal

[2]IFIMUP and IN-Institute of Nanoscience and Nanotechnology, Departamento de Física e Astronomia, Faculdade de Ciências da Universidade do Porto, Rua do Campo Alegre 687, 4169-007 Porto, Portugal

[3]Departamento de Física, Universidade Federal de Santa Catarina, Campus Trindade, 88040-900 Florianópolis, SC, Brazil

[4]Department of Physics, Madanapalle Institute of Technology & Science, Madanapalle-517325, Andhra Pradesh, India

[5]Department of Physics, Central University of Tamil Nadu, Thiruvarur-610 101, India

* E-mail: josesilva@fisica.uminho.pt

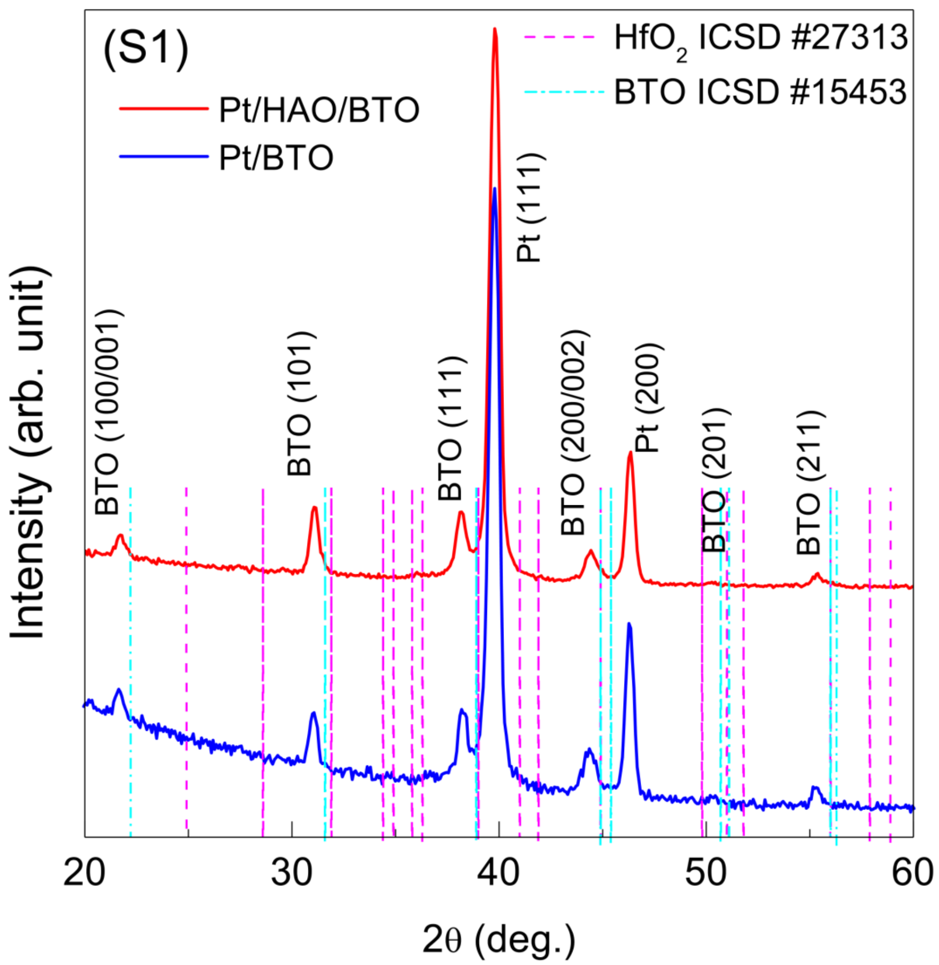

Figure S1. XRD patterns of the Pt/BTO and Pt/HAO/BTO structures.

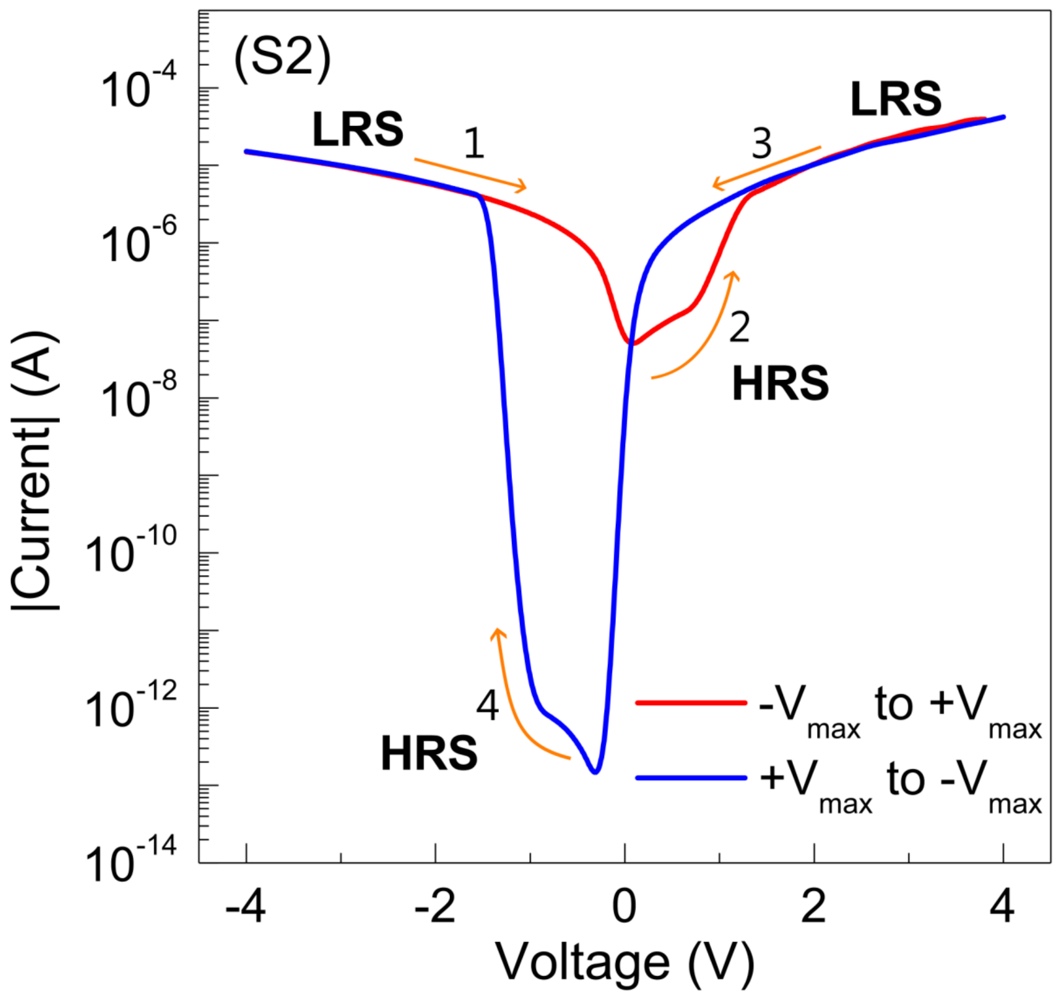

Figure S2. I-V curve with V_{max} = 4 V for the Pt/HAO/BTO/ITO structure. The red curve was measured on forward sweep and the blue curve was measured on reverse sweep.

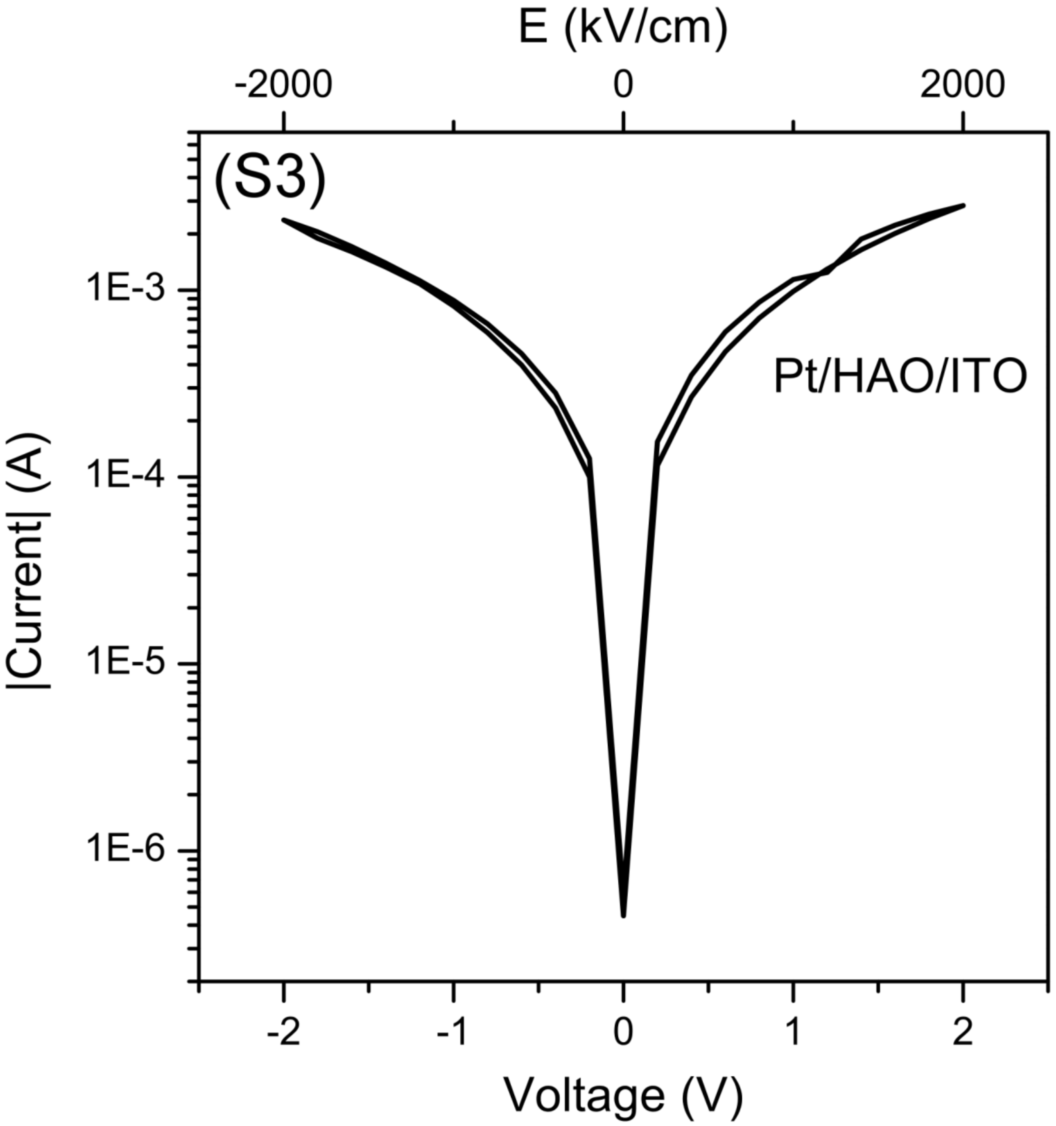

Figure S3. I-V curve for the Pt/HAO (10nm)/ITO structure.
